# The COVID-19 pandemic as a pivot point for biological conservation

**DOI:** 10.1038/s41467-021-25399-5

**Published:** 2021-08-30

**Authors:** Amanda E. Bates, Sangeeta Mangubhai, Celene B. Milanés, Ku’ulei Rodgers, Valeria Vergara

**Affiliations:** 1 Department of Biology, University of Victoria, Victoria, BC Canada; 2 Wildlife Conservation Society, Fiji Country Program, Suva, Fiji; 3 Civil and Environmental Department, Universidad de la Costa, Barranquilla, Atlántico Colombia; 4 University of Hawai’i, Hawai’i Institute of Marine Biology, Coral Reef Ecology Laboratory, Kane’ohe, HI USA; 5 Ocean Wise Conservation Association, Vancouver, BC Canada

**Keywords:** Conservation biology, Restoration ecology

## Abstract

The COVID-19 lockdown reduced human mobility and led to immediate insights into how humans impact nature. Yet the strongest ecological impacts are likely to come. As we emerge from the pandemic, governments should avoid prioritizing short-term economic gains that compromise ecosystems and the services they provide humanity. Instead, the pandemic can be a pivot point for societal transformation to value longer term ecosystem and economic sustainability.

## Impacts of the COVID-19 pandemic on biological conservation

The COVID-19 pandemic has led to shifts in human activities and mobility patterns that have altered all aspects of society. Unexpected opportunities to examine relationships between humans and nature have arisen. Initial findings point to diverse direct and indirect pathways linking shifts in human presence and activity to both positive and negative outcomes for wildlife, ecosystems, and conservation.

For example, the International Quiet Oceans Experiment has encouraged worldwide monitoring of our oceans’ soundscapes to measure how the pandemic-related reduction in shipping and other marine activities affects noise levels, and subsequently ocean ecosystems, from zooplankton to large whales^1^. The lockdown has illuminated the need to set global guidelines and adopt quieting technologies to “turn down the noise”.

Yet as we move to a postpandemic world, some countries are reducing their environmental management and safeguards, with natural resources viewed as “capital” to build economic recovery plans. Thus the pandemic is revealing emerging challenges that require innovative solutions and new ways of working that can enhance efforts to sustain healthy ecosystems and support human well-being.

## Emerging challenges

While the roll-out of vaccines for COVID-19 is presently underway, the ecological, social, and economic legacy of this event will persist. It became immediately apparent that impacts from the pandemic lockdown would be permanent gaps in environmental monitoring and conservation programs^2^. Indeed, the widespread global scale of the event emphasized many challenges.

Policy gaps are prolific and governments lack capacity to react adaptively to multiple disturbances and emerging threats. For example, masks and single-use plastic waste have increased due to the wearing of personal protective equipment against COVID-19. This problem has highlighted the need to unify fragmented authorities governing plastic production and coordinate policies aiming to control plastic pollution, including regulations to the plastic industry and promoting the re-entry of plastic waste into economic circuits^3,4^.

Multiple crises resulted from cumulative and interacting impacts of the COVID-19 pandemic^5^. In the Caribbean and Pacific regions several cyclones caused widespread damage, which diverted limited government funding to emergency relief efforts, and created new challenges that include addressing overlapping humanitarian crises with borders closed and restrictions on movement (e.g., refs. ^5,6^). The capacity for human systems to remain resilient or buffer the impacts of these extreme events has led to great damage in already vulnerable ecosystems. Just prior to the onset of a national lockdown, civil society organizations in Argentina had launched a key initiative to stop the deforestation of the Chaco, the second largest forest ecosystem in South America. But lockdown measures stifled oversight on the ground, with illegal extraction intensifying and fires breaking all records^7^.

Short-sighted decisions are being made as the world enters economic uncertainty and policy is required to recover communities following natural disasters. While livelihoods are naturally at the forefront, this lack of vision is leading to economic drawdown and unregulated resource use, with strong negative impacts on natural systems including biodiversity losses that will impact economic sustainability in the future. Developing countries, the Global South, and Small Island Developing States, whose economies are based on their natural resources, may face a greater risk of decisions which may ultimately harm both humans and wildlife, such as large-scale logging to produce wood products^8,9^. For instance, seasonal grouper bans in Fiji meant to protect spawning populations were lifted early to allow fishers to harvest and sell these species, despite declining populations prepandemic^6^. This reality contrasts starkly with the potential for the pandemic to offer a pivot point for societal transformation. Both ecosystem and economic sustainability are possible if measures are implemented that shift away from activities that damage ecosystems in favor of those which promote resilience^10^. In fact, the pandemic offers potential for societal transformation to promote a longer-term vision for both ecosystem and economic sustainability.

## Novel conservation approaches and solutions that have emerged from the pandemic

The global COVID-19 pandemic has highlighted how changes in the scope, types, and scales of human activities impact biological conservation. More subtle wildlife responses to disturbances need to be considered. Human activities, for example, which may have previously appeared relatively benign (such as hiking and snorkeling), may discourage animals from using their preferred habitats^11^. Limited access to preferred areas for foraging, avoiding predators, or thermoregulation may have important energetic impacts that in turn may influence whether an animal will survive exposure to disease or starvation. Thus, strategies which more explicitly minimize human–wildlife interactions may improve conservation outcomes. Indeed, the negative effects of breaks in programs to protect nature provide strong support for the value of conservation strategies already in place, e.g., programs to eradicate invasive predators or support habitat enrichment of endangered species^11^.

Conservation activities have also adapted, and in some cases may be more successful. For instance, use of dogs for tracking and surveying species was prioritized and possible under the lockdown, simply because this minimizes the number of people required for field work^12^. In Hawai’i, the pandemic reset visitor impacts to zero, prompting better natural resource management funded through user fees, extended breaks, and visitor limitations once tourism resumed, as instituted at the Hanauma Bay Nature Preserve. In light of the pandemic’s high unemployment and loss of businesses, Hawai’i is beginning to reconsider its overdependence on tourism as a primary economic driver^13^.

It has also become even clearer that local stewardship, including Indigenous management systems, and self-reliance are the backbone of successful programs to support conservation at local and global scales. Although comprising less than 5% of the global population, Indigenous Peoples have tenure rights to some of the most intact habitats and ecosystems on this planet^14^. These include areas of intact forests that are crucial to tackling global biodiversity loss and climate change crises. Rates of forest loss have been considerably lower on Indigenous Peoples’ lands than on other lands, although these forests are still vulnerable to clearing and other threats^15^. Mechanisms need to be put in place to ensure Indigenous rights and management systems are not at risk during the COVID-19 pandemic but are instead supported to ensure healthier ecosystems for future generations.

Many scientists have changed the way they work during the lockdown, shifting to virtual meeting platforms to connect with local experts to achieve research goals. As a result of border closures there has been a shift towards a less “extractive” model in research practices. Scientists often “parachute” into countries and into communities to collect specimens and data, leaving nothing of value behind, but also missing opportunities to gain from local natural history and knowledge^16^. This approach has not been possible during the lockdown, and instead external scientists have needed to work remotely via field operations executed by local scientists and community experts. For example, researchers from Dalhousie and Memorial Universities in Atlantic Canada (including co-author Bates) partnered with the Nunatsiavut Government (regional Inuit government) to co-develop and co-lead a research project on sustainable ocean systems^17^. Part of the project’s response to COVID-19 related lockdown protocols was to hire four local Inuit Research Coordinators in different Nunatsiavut communities (rather than just one) to conduct and lead research during the lockdown period, such as deploying instruments through the ice for measuring ocean conditions. Creating a network of community-based positions has now been recognized as invaluable for the success and co-development of the project outputs, and will continue for the life of the project. Indeed, the pandemic response has generally accelerated the recognition that local research teams have locally relevant local knowledge and field expertise, combined with the skills to lead and conduct research in collegial partnerships with scientists based elsewhere.

## Strategies to ensure positive impacts are recognized

**Fig. 1:**
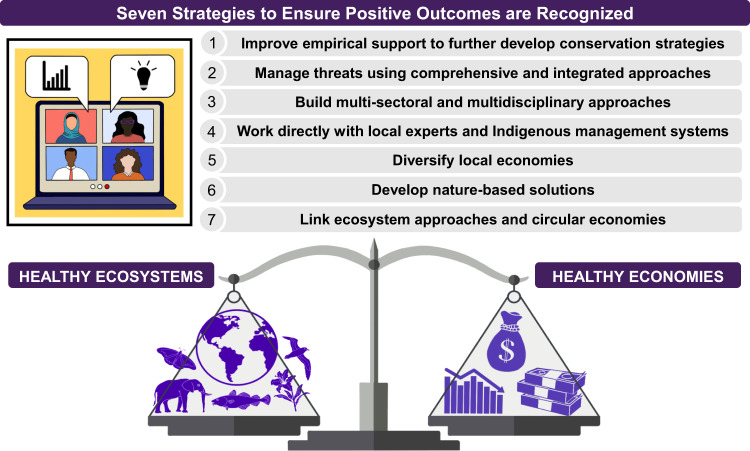
Seven strategies to maximize the COVID-19 pandemic as a pivot point for biological conservation. Societal transformation will promote a longer-term vision for both ecosystem and economic sustainability. Drawings were provided by Cerren Richards.

The entire world has responded to and been impacted by the COVID-19 pandemic. Humans have changed our activities and behaviors, illustrating that rapid societal change is possible. It is important to recognize that many of the root causes of this pandemic are the same as those that are worsening the global climate change and biodiversity crises. As we learn and adapt from this pandemic, opportunities for societal transformation that could change the world and the health of natural systems should not be missed. Vision is needed by our world leaders and those of influence now more than ever to rise from the pandemic years with pathways towards greater sustainability. We suggest seven strategies to maximize the COVID-19 pandemic as a pivot point for biological conservation (Fig. 1).

New understanding gained through the pandemic can be incorporated into conservation plans moving forwards, which will take careful and insightful planning (Fig. 1(1)). This includes fine-tuning predictive models and conservation theory with greater skill and precision. For instance, confining humans to their residences at such large scales has underpinned estimates of the causal impact of reducing human activity on wildlife around the world^11^.

Multiple disturbances and threats are increasing in frequency and intensity (e.g., pandemics, biodiversity loss, climate change). New methodologies with a multi-hazard risk perspective are required (Fig. 1(2)). We call for improvements to management models and prognostic tools to analyze and quantify vulnerabilities across ecological, social, and economic systems in future postpandemic scenarios, coupled with investments to build resilience in these diverse systems to multiple disturbances. Doing so will improve risk management before, during, and after disturbances, including those that overlap, and shift to a more preventative rather than reactive approach.

Solutions need to be multisectorial and coordinated, rather than sacrificing one sector for another (Fig. 1(3)). Strategies can be designed and tested for decision-making to balance short-term gains versus investing in long-term transformations. This involves leveraging multidisciplinary knowledge, expertise, and resources toward a shared goal of producing better environmental and human well-being outcomes.

Partnerships with local experts can support shared-conservation agendas to achieve both sustainable ecosystems and human well-being (Fig. 1(4)). Investing in local community experts and stewardship also has potential to build stronger local economies and long-term capacity. This requires development of the appropriate legislation and policies and adequate allocation of resources (especially funding) to support Indigenous Peoples and communities to participate and lead conservation efforts. For instance, support of local conservation efforts (e.g., expansion of Hawai’i’s Community Based Subsistence Fishing Areas) and inclusion of Indigenous management systems, are being collaboratively supported by Indigenous Peoples, local communities, governmental and non-governmental organizations, and scientists worldwide.

Regions, which heavily and narrowly rely on funding from a single sector (such as international tourism) to support biodiversity conservation, are vulnerable to external shocks and require diversification. This is fundamental for economic resilience and protection against global crises such as pandemics (Fig. 1(5)). Diversification of local economies may offer viable alternatives to (over)exploitation or illegal and unregulated resource use.

Strong links between environmental and human health have also come to light (“One Health”) that reinforce support of conservation programs and nature-based solutions^18^. This needs to be better reflected in policies, strategies, and action from global to local levels. Linking conservation of nature to human health may dampen economic drawdown and lead to strong human well-being and conservation outcomes (Fig. 1(6))

Social, economic, and biological systems are intimately connected. We urge economists to engage with ecologists (and vice versa) in discussions about how ecosystem valuation can strengthen the relationship between sustainable development, nature, and society (Fig. 1(7)).
